# The burden of choice: decision regret in subspecialty selection among young and middle-aged doctors

**DOI:** 10.1186/s12909-026-08858-9

**Published:** 2026-02-24

**Authors:** Peixuan Guan, Maosha Fu, Feng Wu, Liru Hou, Weiyu Zhang

**Affiliations:** 1 Department of Continuing Education, Peking University People’s Hospital, Beijing, China; 2 Department of Human Resources, Peking University People’s Hospital, Beijing, China; 3 Chinese PLA center for disease control and prevention, Beijing, China; 4 Department of Urology, Peking University People’s Hospital, Beijing, China

**Keywords:** Decision regret, Subspecialty selection, Burnout, Job satisfaction, Psychological resilience

## Abstract

**Background:**

Subspecialty choice plays a pivotal role in physicians’ career satisfaction and professional trajectory. However, decision regret regarding subspecialty selection has been underexplored, despite its potential impact on well-being and retention.

**Methods:**

A cross-sectional study was conducted among 615 young and middle-aged physicians (aged 25–55) from secondary and tertiary hospitals in China. Participants completed an online questionnaire including the Decision Regret Scale (DRS), a simplified Maslach Burnout Inventory (MBI), Minnesota Satisfaction Questionnaire (MSQ), and Connor-Davidson Resilience Scale (CD-RISC). Statistical analyses involved ANOVA, Spearman’s correlation, and multivariate linear regression to identify factors associated with decision regret.

**Results:**

Among participants, 9.4% reported high regret, 37.6% moderate regret, and 53.0% low regret. Higher regret was significantly associated with male gender (*P* = 0.039), younger age (*P* = 0.019), and higher professional title (*P* = 0.003). Regression analysis revealed that greater burnout (B = 1.521, *P* < 0.001) predicted higher regret, while higher job satisfaction (B = − 0.339, *P* < 0.001) and resilience (B = − 0.588, *P* = 0.001) were protective factors.

**Conclusion:**

Nearly one in five young and middle-aged Chinese physicians experience high regret in subspecialty choice, influenced by gender, age, burnout, satisfaction, and resilience. Targeted interventions addressing these factors may mitigate regret and enhance physician well-being and retention.

**Supplementary Information:**

The online version contains supplementary material available at 10.1186/s12909-026-08858-9.

## Introduction

Choosing a subspecialty is a defining step for physicians, shaping career development, fulfillment, and service delivery. Previous studies have shown that well-aligned specialty choices enhance professional satisfaction and reduce burnout by matching individual interests with long-term goals [1, 2]. Subspecialty training also fosters clinical competence and the ability to meet evolving healthcare needs such as aging populations and chronic disease management [3, 4]. In this process, factors such as lifestyle expectations, mentorship, and institutional structure play significant roles in shaping doctors’ decisions [5–7].

However, an increasing number of physicians report regret regarding their subspecialty choice, a psychological experience that may adversely affect well-being and retention [8]. Decision regret in medicine has traditionally been studied in the context of clinical decisions, but less is known about how it manifests in career decisions. Studies from the United States and Europe indicate that career choice regret correlates with burnout, low satisfaction, and mental distress among doctors in training [9–12]. Furthermore, those dissatisfied with their subspecialty choice are less likely to pursue leadership roles or academic advancement [1]. In contrast, little systematic evidence exists from non-Western settings, where cultural norms, rigid training pathways, and limited flexibility may heighten the emotional consequences of early career decisions [13, 14].

Most existing literature focuses on job satisfaction or burnout rather than the distinct emotion of regret—a backward-looking evaluation of one’s past choices [15]. Furthermore, validated instruments such as the Decision Regret Scale (DRS) have rarely been applied to physicians’ career contexts [16, 17]. This gap limits understanding of the psychological impact of specialty decisions, particularly in healthcare systems where opportunities for re-specialization are constrained.

Therefore, this study aims to quantify decision regret among young and middle-aged physicians in China and identify associated psychological and demographic factors. By situating this analysis within a global research context, we seek to contribute to the growing cross-cultural understanding of how career decision-making influences physician well-being and retention.

## Methods

### Study design and participants

This was a retrospective, cross-sectional study conducted among young and middle-aged physicians working in secondary or tertiary hospitals in China. A total of 615 physicians were recruited through purposive sampling. This method was chosen to ensure inclusion of physicians with completed subspecialty training and diverse clinical backgrounds, thereby capturing a broad range of experiences related to decision regret. However, we acknowledge that purposive sampling may limit generalizability due to potential selection bias; thus, findings should be interpreted with caution and confirmed by future studies using probability sampling methods. Inclusion criteria were: (1) aged 25–55 years, (2) having completed subspecialty selection and working in their chosen field for at least one year, and (3) currently employed in a secondary or tertiary hospital. Physicians who had left clinical practice for more than one year or declined participation were excluded. The study protocol was approved by the Ethics Committee of Peking University People’s Hospital.

### Data collection

Participants were invited to complete an online structured questionnaire. Data were collected on demographic and professional characteristics, including age, gender, educational background, professional title, current hospital level (secondary or tertiary), subspecialty initially selected, current subspecialty, and whether they remained in clinical practice.

### Measures

#### Decision regret

Decision regret was assessed using the Decision Regret Scale (DRS) [18], a validated five-item instrument measuring regret after healthcare-related decisions, adapted for subspecialty selection in this study. Given that the participants in this study were all from China, we used this Chinese version to assess decision regret related to their choice of subspecialty, with a Cronbach’s α coefficient of 0.826 [19]. Scores ranged from 0 to 100, with higher scores indicating greater regret. Based on the scores of the DRS, the participants were divided into three groups: high regret (score > 66), moderate regret (score between 34 and 66), and low regret (score < 34). The adaptation process followed forward–backward translation by two bilingual experts, followed by expert panel review to ensure semantic equivalence and content validity. A pilot test involving 30 physicians was conducted to assess clarity and internal consistency. The adapted DRS demonstrated good reliability (Cronbach’s α = 0.87).

#### Bournut

The original MBI’s reliability and validity in assessing occupational burnout provided a foundation for evaluating post-decision regret, as emotional exhaustion, detachment, and eroded self-worth are similarly salient in decision-related distress. To evaluate decision regret among participants, we adapted a concise measurement tool based on the MBI with reference to previous research designs [20, 21], a validated instrument assessing burnout across three key dimensions. Our simplified scale retains the core constructs of the MBI while focusing on items most relevant to post-decision emotional and cognitive responses. Pilot testing in the same 30-physician sample yielded satisfactory internal consistency (Cronbach’s α = 0.82) and confirmed face validity. The subscales are seen in supplementary materials.

#### Job satisfaction

Job satisfaction was evaluated with the short form of the Minnesota Satisfaction Questionnaire (MSQ), covering two dimensions: Intrinsic Satisfaction and Extrinsic Satisfaction. Higher scores indicated greater satisfaction.

#### Psychological resilience

Psychological resilience was measured by the Connor-Davidson Resilience Scale (CD-RISC), including three dimensions: Self-confidence and Sense of Control, Emotional Management and Stress Coping, and Social Support and Goal Orientation.

### Handling of missing data and sensitivity analysis

Data were screened for missing values. Responses with more than 10% missing items were excluded (*n* = 12). For questionnaires with fewer than 10% missing responses, missing values were imputed using the series mean method. To assess the robustness of results, we conducted sensitivity analyses excluding imputed cases, which yielded consistent statistical outcomes, supporting the stability of the findings.

### Statistical analysis

Descriptive statistics were used to summarize demographic and professional characteristics. Differences in shortened MBI, MSQ, and CD-RISC subscale scores across DRS tertiles were examined using ANOVA or Kruskal-Wallis tests, as appropriate. Spearman’s rank correlation analysis was performed to explore the associations between DRS scores and continuous variables from shortened MBI, MSQ, and CD-RISC. Multivariate linear regression was conducted to identify risk factors associated with higher levels of decision regret. Further stratified analyses were performed to explore potential reasons for regret based on participants’ current clinical status (i.e., whether they remained in clinical practice and whether they remained in their originally selected subspecialty).

All statistical analyses were performed using IBM SPSS Statistics (version 22.0; IBM Corp., Armonk, NY, USA), with a two-tailed significance level of *p* < 0.05.

## Results

### General characteristics of clinical doctors participating in the study

A total of 615 physicians were included in the study (Table 1). The majority were male (60.7%) and aged 30–34 years (46.8%). Most held a doctoral degree (51.4%) and worked as attending physicians (45.4%). Over half specialized in surgery (53.5%), and 89.4% remained in clinical practice. The mean score of DRS was 34.9 ± 23.2 and the scores of other questionnaires were displayed in Table 2. This shows that most clinical doctors adhere to the front - line of clinical work and their initially chosen professional positions, but there is still a certain proportion of personnel changes. In this study on the decision - regret situation of clinical doctors after choosing their secondary disciplines, multiple questionnaires were employed to assess the psychological states and work - related perceptions of clinical doctors. The comprehensive analysis of the scores from these questionnaires provides comprehensive and crucial data support for in - depth exploration of various states of clinical doctors after choosing their secondary disciplines.

**Table 1:** General characteristics of participating physicians

Items	*N* (%)
In all	615
Gender	
Male	373 (60.7%)
Female	242 (39.3%)
Age	
<30	143 (23.3%)
30–34	288 (46.8%)
35–39	133 (21.6%)
≥40	51 (8.3%)
Academic degree	
Undergraduate graduate	71 (11.5%)
Master’s degree graduate	202 (32.8%)
Doctoral degree graduate	316 (51.4%)
Post-doc	26 (4.2%)
Professional titles	
Physician	182 (29.6%)
Attending Physician	279 (45.4%)
Associate Chief Physician	66 (10.7%)
Chief Physician	12 (2.0%)
Unrated professional title	76 (12.4%)
Secondary discipline	
Internal medicine	103 (16.7%)
Surgery	329 (53.5%)
Obstetrics and Gynecology	28 (4.6%)
Anesthesiology	27 (4.4%)
Others	126 (20.5%)
Are you still engaged in clinical work?	
Yes	550 (89.4%)
No	65 (10.6%)
Are you still working in the major that you chose for the first time?	
Yes	479 (77.9%)
No	71 (11.5%)

**Table 2:** Mean scores of psychological and work-related questionnaires

Items	Score (mean ± SD)
Decision regret scale	34.9 ± 23.2
Maslach burnout inventory	
In total	22.4 ± 6.2
Emotional exhaustion	11.2 ± 3.1
Depersonalization	8.8 ± 3
Personal accomplishment	2.4 ± 0.9
Minnesota satisfaction questionnaire	
In total	60.7 ± 11.8
Intrinsic satisfaction	34.4 ± 6.6
Extrinsic satisfaction	26.3 ± 5.7
Connor - Davidson Resilience Scale	
In total	36 ± 6.1
Self - confidence and sense of control	10.8 ± 1.9
Emotional management and stress coping	14.3 ± 2.6
Social support and goal - orientation	10.9 ± 2

### Analysis of factors associated with decision

The decision - regret situation among clinical doctors after choosing their secondary disciplines was analyzed. Based on the Decision Regret Scale, participants were grouped as high regret (9.4%, 58 cases), moderate regret (37.6%, 231 cases), and low regret (53.0%, 326 cases). Spearman’s rank-correlation analysis revealed: 53.4% (31 cases) of high-regret subjects were male, 46.6% (27 cases) female (*P* = 0.039); 37.9% (22 cases) of high-regret subjects were < 30 years (*P* = 0.019); academic degree (*P* = 0.351) and secondary discipline (*P* = 0.088) showed no significance, while professional title was correlated (*P* = 0.003). Gender, age, and professional title were significantly associated with decision regret; academic degree and secondary discipline were not (Table 3).

**Table 3:** Demographic and professional characteristics by decision regret level

	Decision regret scale			*P* value∮
	High (>66)	Moderate (34–66)	Low (<34)	
In all	58 (9.4%)	231 (37.6%)	326 (53.0%)	
Gender				0.039*
Male	31 (53.4%)	129 (55.8%)	213 (65.3%)	
Female	27 (46.6%)	102 (44.2%)	113(34.7%)	
Age				0.019*
<30	22 (37.9%)	54 (23.4%)	67 (20.6%)	
30–34	28 (48.3%)	108 (46.8%)	152 (46.6%)	
35–39	6 (10.3%)	45 (19.5%)	82 (25.2%)	
≥40	2 (3.4%)	24 (10.4%)	25 (7.7%)	
Academic degree				0.351
Undergraduate graduate	5 (8.6%)	31 (13.4%)	35 (10.7%)	
Master’s degree graduate	22 (37.9%)	84 (36.4%)	96 (29.4%)	
Doctoral degree graduate	28 (48.3%)	106 (45.9%)	182 (55.8%)	
Post-doc	3 (5.2%)	10 (4.3%)	13 (4.0%)	
Professional titles				0.003**
Physician	23 (39.7%)	60 (26.0%)	99 (30.4%)	
Attending Physician	18 (31.0%)	100 (43.3%)	161 (49.4%)	
Associate Chief Physician	4 (6.9%)	30(13.0%)	32 (9.8%)	
Chief Physician	0 (0%)	4 (1.7%)	8 (2.5%)	
Unrated professional title	13 (22.4%)	37 (16.0%)	26 (8.0%)	
Secondary discipline				0.088
Internal medicine	11 (19.0%)	44 (19.2%)	48 (14.7%)	
Surgery	25 (43.1%)	113 (49.3%)	191 (58.6%)	
Obstetrics and Gynecology	6 (10.3%)	10 (4.4%)	12 (3.7%)	
Anesthesiology	2(3.4%)	13 (5.7%)	12 (3.7%)	
Others	14 (24.2%)	49(21.4%)	63 (19.3%)	

∮, Spearman’s rank correlation analysis, * *P* < 0.05, ** *P* < 0.01, *** *P* < 0.001

### Decision regret correlates

Distribution of decision regret among young and middle-aged physicians across different demographic and professional characteristics was displayed in Table 3. Among the 615 respondents, 9.4% reported high regret, 37.6% moderate regret, and 53.0% low regret.

Gender differences showed that a higher proportion of males experienced high regret (53.4%) compared to females (46.6%), with a marginally significant correlation (*P* = 0.039). Age was inversely correlated with regret (*P* = 0.019), with the 30–34 age group comprising the largest share of high regret (48.3%). Academic degree showed no significant correlation with regret (*P* = 0.351).

Regarding professional titles, physicians were most represented in the high regret group (39.7%), followed by attending physicians (31.0%, *P* = 0.003). Subspecialty distribution revealed that surgeons accounted for 43.1% of high regret cases, internal medicine for 19.0%, while no significant correlation was found (*P* = 0.088).

### Regret risk factors

The results of a multivariate regression analysis examining factors associated with decision regret among young and middle-aged physicians in subspecialty selection was presented in Table 4. Independent variables included dimensions from the shortened MBI, the MSQ, and the CD-RISC.

**Table 4:** Multivariate regression analysis of factors associated with decision regret

Model		Unstandardized coefficient		Standardized coefficient	t	Significance	95.0% confidence interval of B	
		B	Standard error	Beta			Lower limit	Upper limit
MBI	In total	1.521***	0.156	0.408	9.728	0.000	1.214	1.828
	MBIEE	2.477***	0.344	0.332	7.196	0.000	1.801	3.153
	MBIDP	0.256	0.345	0.033	0.742	0.458	-0.422	0.934
	MBIPA	3.199***	1.128	0.130	2.837	0.005	0.985	5.414
MSQ	In total	-0.339***	0.083	-0.173	-4.09	0.000	-0.502	-0.176
	Internal satisfaction	-1.001***	0.213	-0.287	-4.700	0.000	-1.419	-0.583
	External satisfaction	-0.982***	0.247	-0.243	-3.981	0.000	-1.466	-0.498
CDRISC	In total	-0.588***	0.175	-0.156	-3.361	0.001	-0.932	-0.245
	Self-confidence and sense of control	-0.886	0.982	-0.073	-0.902	0.367	-2.814	1.042
	Emotional management and stress coping	-2.121***	0.689	-0.236	-3.079	0.002	-3.475	-0.768
	Social support and goal orientation	-3.022***	0.756	-0.262	-3.998	0.000	-4.507	-1.538

*Abbreviations: DRS* Decision Regret Scale, *MBI* Maslach Burnout Inventory, *MSQ* Minnesota Satisfaction Questionnaire, *CD-RISC* Connor-Davidson Resilience Scale, *EE* Emotional Exhaustion, *DP* Depersonalization, *PA* Personal Accomplishment. * *P* < 0.05, ** *P* < 0.01, *** *P* < 0.001

Burnout, as measured by shortened MBI total score, was positively associated with decision regret (B = 1.521, 95% confidence interval [CI]: 1.214–1.828, *P* < 0.001), indicating that higher burnout predicted higher regret. Specifically, MBIEE showed a strong positive association (B = 2.477, 95% CI: 1.801–3.153, *P* < 0.001), while MBIPA was also positively related (B = 3.199, 95% CI: 0.985–5.414, *P* = 0.005). MBIDP was not a significant predictor (*p* = 0.458).

Job satisfaction showed an inverse association with decision regret. The MSQ total score was negatively associated with regret (B = − 0.339, 95% CI: -0.502- -0.176, *P* < 0.001), suggesting that greater job satisfaction reduced regret. Specifically, both internal satisfaction (B = -1.001, 95% CI: -1.419- -0.583, *P* < 0.001) and external satisfaction (B = -0.982, 95% CI: -1.466- -0.498, *P* < 0.001) were significantly associated with lower decision regret, with internal satisfaction having a stronger effect. This indicates that higher levels of both intrinsic and extrinsic satisfaction with one’s job can help reduce the sense of regret related to career decisions.

CD-RISC total was also inversely associated with regret (B = − 0.588, 95% CI: -0.9932- -0.245, *P* = 0.001), indicating that higher resilience reduced regret. Specifically, Emotional management and stress coping (B = -2.121, 95% CI: -3.475 - -0.768, *P* = 0.002) and social support and goal orientation (B = -3.022, 95% CI: -4.507- -1.538, *P* < 0.001) were both significantly associated with lower decision regret, suggesting that better emotional regulation and support networks can reduce regret. However, Self-confidence and sense of control (B = -0.886, 95% CI: -2.814- 1.042, *P* = 0.367) did not show a significant effect, indicating that while overall resilience is protective, specific components may vary in their impact on decision regret.

## Discussion

Based on the study, 9.4% of young and middle-aged physicians reported high decision regret regarding subspecialty selection, with higher regret associated with male gender, younger age, lower job satisfaction, higher burnout, and lower resilience. These findings extend existing research by focusing on the emotional dimension of career decision-making—a topic often overshadowed by studies on satisfaction or burnout.

This prevalence aligns partially with international findings; for example, Western studies have similarly identified burnout and low job satisfaction as predictors of specialty regret, yet report slightly lower rates of regret among early-career physicians [22, 23]. Unlike studies in the U.S. and Europe that found female physicians more likely to experience career regret due to work-life balance conflicts [24], our study observed higher regret among male physicians, which may reflect gendered expectations in Chinese medical culture. It is possible that men, influenced by traditional societal norms, experience stronger pressures for financial and professional success, which could amplify feelings of regret when career trajectories do not align with these expectations. Studies on gender roles in Chinese culture suggest that men may face greater societal pressure to succeed professionally and financially, which may heighten self-blame and regret when their career choices do not meet those expectations [25]. Moreover, the lack of significant correlation between academic degree or subspecialty type and regret contrasts with international research highlighting specialty-specific burnout risk (e.g., higher in emergency medicine or surgery) [26], suggesting possible differences in medical training structure and workload distribution across healthcare systems. In the Chinese context, this reversal may be interpreted through a sociocultural lens. Traditional gender roles and Confucian values in Chinese culture emphasize men’s responsibility for professional and financial success, which may contribute to heightened self-blame and regret when their career outcomes do not meet these expectations. Previous studies have suggested that gender roles, particularly in Confucian-influenced cultures, can place significant pressure on men to succeed professionally, which may increase their susceptibility to regret when career choices diverge from these culturally defined norms [27, 28]. The strong link between burnout and regret in our findings supports prior research identifying burnout as both a cause and consequence of career dissatisfaction [22, 23]. Furthermore, the protective role of resilience parallels findings from Western contexts [29], underscoring the universal value of resilience-building interventions despite differing healthcare systems. In sum, while our study aligns with global evidence linking burnout, low satisfaction, and low resilience to specialty regret, differences in gender patterns and the absence of specialty-specific regret highlight the influence of cultural, systemic, and educational factors unique to the Chinese medical context.

In our study, several variables significantly influenced decision regret among young and middle-aged physicians, including gender, age, job satisfaction, burnout, and psychological resilience. Male physicians reported higher regret, which may reflect gender-based societal expectations in China that place greater pressure on men to succeed professionally and financially, amplifying dissatisfaction when career alignment is lacking [30, 31]. Younger physicians experienced more regret, which may stem from limited exposure to diverse specialties during training. This limitation might lead to premature decisions influenced by external factors such as mentors or institutional demands, as suggested in previous studies of early career decision-making in healthcare professionals [32]. Low job satisfaction was strongly associated with higher regret, which may suggest that when professional fulfillment and intrinsic motivation are lacking, physicians could retrospectively view their specialty choice as misaligned with their personal values or lifestyle goals. Similar associations have been observed in other studies on career regret among healthcare professionals, highlighting the importance of aligning career choices with individual needs and motivations [33, 34]. High burnout, especially emotional exhaustion, intensified regret, likely because chronic stress undermines one’s ability to find meaning in work, making previous decisions seem erroneous [35]. Conversely, greater psychological resilience appeared protective, as it enhances coping, optimism, and adaptability, reducing the tendency to dwell on past decisions negatively [36].

Our study shows that decision regret in subspecialty selection significantly affects physicians’ career well-being by increasing burnout, reducing job satisfaction, and elevating turnover intentions. Physicians with higher regret reported more emotional exhaustion and lower personal accomplishment, suggesting that regret weakens motivation and engagement [32]. Similar studies have found that regret contributes to emotional withdrawal and dissatisfaction, which may increase the risk of leaving clinical practice [33]. Given these risks, early career interventions such as structured career planning, mentorship, and psychological support are critical. These interventions could help young physicians make more informed decisions, reduce regret, and build resilience [37]. Similar interventions have been shown to be effective in Western contexts, and adapting these approaches to the Chinese cultural context may further support the well-being of young doctors. Such support may buffer the negative effects of regret and promote long-term professional satisfaction.

Based on our findings, we recommend early interventions to reduce decision regret and enhance career satisfaction among physicians. Career counseling should be introduced during medical school and residency to guide specialty choices aligned with personal goals, reducing misalignment and regret. Expanding rotational experiences across specialties can provide broader exposure and better decision-making foundations. Establishing mentorship programs to connect trainees with senior physicians can offer personalized guidance and emotional support, helping to mitigate regret and build resilience. Additionally, integrating well-being and career development education into training can lower burnout risk and improve physician retention.

Although innovative, this study has several limitations. First, the cross-sectional design precludes causal inference between decision regret and its associated factors; longitudinal studies are needed to clarify temporal relationships. Second, because data were collected through an online self-reported survey, self-selection bias may have occurred—physicians with stronger emotions or higher regret might have been more likely to participate, potentially affecting representativeness. Third, the sample was drawn only from secondary and tertiary hospitals in China, which may limit generalizability to other healthcare settings or countries. Finally, as all variables were self-reported, recall bias and social desirability bias cannot be entirely ruled out. Future research should employ multi-center, longitudinal, and mixed-method designs to address these limitations and validate our findings across diverse contexts.

In the future research, longitudinal research would be better to track the changes in decision regret over time, more psychological factors (such as perfectionism, coping style) would be worthy to explore, and comparative research in different countries and cultural backgrounds would provide more insights into the decision regret in subspecialty selection.

## Conclusion

By integrating psychological, cultural, and institutional perspectives, this study highlights decision regret as an important yet overlooked dimension of physician well-being. Addressing this issue through early guidance, mentorship, and mental health support can enhance satisfaction, resilience, and long-term retention within the healthcare workforce.

## Supplementary Information

Supplementary Material 1.

## Data Availability

The data are available from the corresponding author upon reasonable request.

## Abbreviations

DRS: Decision Regret Scale
MBI: Maslach Burnout Inventory
MSQ: Minnesota Satisfaction Questionnaire
CD-RISC: Connor-Davidson Resilience Scale
EE: Emotional Exhaustion
DP: Depersonalization
PA: Personal Accomplishment

## References

[1] National Academies of Sciences, Engineering, and Medicine. The Future Pediatric Subspecialty Physician Workforce: Meeting the Needs of Infants, Children, and Adolescents. Washington, DC: The National Academies Press. 2023. 38295208

[2] Jain S, Dewey RS. The Role of "Special Clinics" in Imparting Clinical Skills: Medical Education for Competence and Sophistication. Adv Med Educ Pract. 2021;12:513–8. 10.2147/AMEP.S306214. 10.2147/AMEP.S306214 PMC8148655 34045915

[3] Michalik B, Kulbat M, Domagała A. Factors affecting young doctors’ choice of medical specialty—A qualitative study. PLoS ONE. 2024;19(2):e0297927. 38300924 10.1371/journal.pone.0297927 PMC10833556

[4] Bates T, Kottek A, Spetz J. Geriatrician roles and the value of geriatrics in an evolving healthcare system. San Francisco, CA: UCSF Health Workforce Research Center on Long-Term Care; 2019.

[5] Pham HH, et al. Financial pressures spur physician entrepreneurialism. Health Aff. 2004;23(2):70–81. 10.1377/hlthaff.23.2.70 15046132

[6] Almajdi ALA, et al. The impact of continuing education on the efficiency and performance of healthcare practitioners across different specialties. Gland Surg. 2024;9(2):332–40.

[7] Mbata AO, et al. Preventative Medicine and Chronic Disease Management: Reducing Healthcare Costs and Improving Long-Term Public Health. Int J Multidisciplinary Res Growth Evaluation. 2024;5(06):1584–600.

[8] Kupis R, Michalik B, Polak M, Kulbat M, Domagała A. Specialty choices among new generation of doctors - insights from a Polish survey study. Sci Rep. 2024;14(1):27855. 10.1038/s41598-024-79079-7. 10.1038/s41598-024-79079-7 PMC11561056 39537818

[9] Yang L, et al. Relationship between job burnout, depressive symptoms, and career choice regret among Chinese postgraduates of stomatology. Int J Environ Res Public Health. 2022;19(23):16042. 36498119 10.3390/ijerph192316042 PMC9740178

[10] Hanna KF, Koo K. Professional burnout and career choice regret in urology residents. Curr Urol Rep. 2024;25(12):325–30. 39017800 10.1007/s11934-024-01226-4

[11] Lu DW, et al. Workplace mistreatment, career choice regret, and burnout in emergency medicine residency training in the United States. Ann Emerg Med. 2023;81(6):706–14. 36754699 10.1016/j.annemergmed.2022.10.015

[12] Hodkinson A, Zhou A, Johnson J, Geraghty K, Riley R, Zhou A, Panagopoulou E, Chew-Graham CA, Peters D, Esmail A, Panagioti M. Associations of physician burnout with career engagement and quality of patient care: systematic review and meta-analysis. BMJ. 2022;378:e070442. 10.1136/bmj-2022-070442. 10.1136/bmj-2022-070442 PMC9472104 36104064

[13] Budjanovcanin A, Woodrow C. Regretting your occupation constructively: A qualitative study of career choice and occupational regret. J Vocat Behav. 2022;136:103743.

[14] Koo K, et al. Professional burnout, career choice regret, and unmet needs for well-being among urology residents. Urology. 2021;157:57–63. 34174271 10.1016/j.urology.2021.05.064

[15] Sierra T, Forbes J, Nelson M. Career regret among physician assistants: a comparative survey of primary and specialty care careers[J]. Internet Journal of Allied Health Sciences and Practice. 2019;17(1):10.

[16] Courvoisier DS, Agoritsas T, Perneger TV, Schmidt RE, Cullati S. Regrets associated with providing healthcare: qualitative study of experiences of hospital-based physicians and nurses. PLoS One. 2011;6(8):e23138. 10.1371/journal.pone.0023138. 10.1371/journal.pone.0023138 PMC3149073 21829706

[17] Diotaiuti P, Valente G, Mancone S, Grambone A, Chirico A, Lucidi F. The use of the Decision Regret Scale in non-clinical contexts. Front Psychol. 2022;13:945669. 10.3389/fpsyg.2022.945669. 10.3389/fpsyg.2022.945669 PMC9520623 36186382

[18] Brehaut JC, et al. Validation of a decision regret scale. Med Decis Mak. 2003;23(4):281–92. 10.1177/0272989X03256005 12926578

[19] Chen F, Chen X. 中文版决策后悔量表应用于面部美容受术者的信效度评价 J Nursing(China). 2018;25(7):42–4.

[20] Lim WY, Ong J, Ong S, Hao Y, Abdullah HR, Koh DL, Mok USM. The Abbreviated Maslach Burnout Inventory Can Overestimate Burnout: A Study of Anesthesiology Residents. J Clin Med. 2019;9(1):61. 10.3390/jcm9010061. 10.3390/jcm9010061 PMC7020051 31888017

[21] Wang A, Duan Y, Norton PG, Leiter MP, Estabrooks CA. Validation of the Maslach Burnout Inventory-General Survey 9-item short version: psychometric properties and measurement invariance across age, gender, and continent. Front Psychol. 2024;15:1439470. 10.3389/fpsyg.2024.1439470. 10.3389/fpsyg.2024.1439470 PMC11286593 39081375

[22] Hodkinson A, et al. Associations of physician burnout with career engagement and quality of patient care: systematic review and meta-analysis. BMJ. 2022;378:e070442. 36104064 10.1136/bmj-2022-070442 PMC9472104

[23] Nagle E, et al. Factors affecting healthcare workers burnout and their conceptual models: scoping review. BMC Psychol. 2024;12(1):637. 39511697 10.1186/s40359-024-02130-9 PMC11545506

[24] Akanji B, Mordi C, Ajonbadi HA. The experiences of work-life balance, stress, and coping lifestyles of female professionals: insights from a developing country. Empl Relations: Int J. 2020;42(4):999–1015.

[25] Chen Y, Zhang X. Gender differences in relation of gender role attitudes and happiness-a mixed-methods research from China. Front Psychol. 2024;15:1419942. 39346500 10.3389/fpsyg.2024.1419942 PMC11427413

[26] Jain S, Madani KS, Swaroop M. Inaugural Women in Medicine Summit: An Evolution of Empowerment in Chicago, Illinois, September 20 and 21, 2019: Event Highlights, Scientiic Abstracts, and Dancing with Markers. Int J Acad Med. 2019;5(3):240–301.

[27] Xie Z. Regarding men as superior to women: impacts of Confucianism on family norms in China. China Popul Today. 1994;11(6):12–6. 12290499

[28] Zhang Y, Liu H. Individual’s gender ideology and happiness in China. Chin Sociol Rev. 2022;54(3):252–77. 35814530 10.1080/21620555.2021.1871727 PMC9268205

[29] Adeeniyan A, Ifeoma C, Amara E. Enhancing Health Resilience through the Integration of Mindful Practices and Cultural Dimensions. J Asian-african Focus Health. 2024;2(2):52–63.

[30] Djulbegovic M, et al. Thinking styles and regret in physicians. PLoS ONE. 2015;10(8):e0134038. 26241650 10.1371/journal.pone.0134038 PMC4524595

[31] Müller BS, et al. Regret among primary care physicians: a survey of diagnostic decisions. BMC Fam Pract. 2020;21:1–7. 32183738 10.1186/s12875-020-01125-w PMC7079478

[32] Dyrbye LN, et al. Association of clinical specialty with symptoms of burnout and career choice regret among US resident physicians. JAMA. 2018;320(11):1114–30. 30422299 10.1001/jama.2018.12615 PMC6233627

[33] Cheval B, et al. Associations of regrets and coping strategies with job satisfaction and turnover intention: international prospective cohort study of novice healthcare professionals. Swiss Med Wkly. 2019;149:17–8. 10.4414/smw.2019.20074 31026043

[34] Hale B. The Anatomy of Physician Fulfillment: Strategies Beyond Burnout. Antioch University; 2024.

[35] Bajada N. Meaning of Work, Burnout and Career Choice Regret: COVID-19 and Healthcare Workers in Malta. 2021, Universidade NOVA de Lisboa (Portugal).

[36] Xing C, Sun J-m. The role of psychological resilience and positive affect in risky decision-making. Int J Psychol. 2013;48(5):935–43. 23170790 10.1080/00207594.2012.729840

[37] Haberer JE, et al. Mentorship-A critical metric for career development and advancing global health. PLOS Glob Public Health. 2025;5(10):e0005247. 41091681 10.1371/journal.pgph.0005247 PMC12527202

