# Supplementary material for: The burden of choice: decision regret in subspecialty selection among young and middle-aged doctors
Source: BMC Med Educ. 2026 Feb 24;26:520. doi: 10.1186/s12909-026-08858-9 (PMC13037031; doi:10.1186/s12909-026-08858-9)
Supplement: Supplementary file 1 — Supplementary Material 1. [file 12909_2026_8858_MOESM1_ESM.docx]

Description of Psychometric Scales and Questionnaires Used in the Study

**1. Decision Regret Scale (DRS)**

Participants rated their agreement with the following statements on a 5-point Likert scale (1 = Strongly disagree, 5 = Strongly agree):

1. "It was the right decision."

2. "I regret the choice that was made."

3. "I would go for the same choice if I had to do it over again."

4. "The choice did me a lot of harm."

5. "The decision was a wise one."

The scores were standardized to a 0-100 scale using the following formula:

**Percentage Score=(Original Score−5​)/20×100**

This converts the original range, with 5 representing 0% (minimal regret) and 25 representing 100% (maximum regret). The regret levels were categorized into three equal tertiles based on the new 0-100 scale: low regret (0-33%), moderate regret (34-66%), and high regret (67-100%). Given that the participants in this study were all from China, we used this Chinese version to assess decision regret related to their choice of subspecialty [1].

1. 陈芳,程晓姣. 中文版决策后悔量表应用于面部美容受术者的信效度评价[J]. 护理学报,2018,25(7):42-44. DOI:10.16460/j.issn1008-9969.2018.07.042.

**2. Maslach Burnout Inventory (MBI)**

The shortened version of Maslach Burnout Inventory (MBI) scale comprises the following subscales:

**1. Emotional Exhaustion (EE)**

This subscale captures feelings of being emotionally overextended and drained due to one’s role in the decision-making process. Participants rate their agreement (e.g., on a 5-point Likert scale) with statements such as:

- *"I feel extremely fatigued by my work."*
- *"I have no energy left to cope with my responsibilities."*
- *"My work has left me feeling completely exhausted."*
- *"I have lost interest in my work."*

**2. Depersonalization (DP)**

This subscale measures the development of detached or impersonal attitudes toward others affected by the decision. Example items include:

- *"I feel indifferent or emotionally distant toward people I work with."*
- *"I no longer care about the needs of clients or stakeholders."*
- *"I have become emotionally hardened in my role."*
- *"At times, I react impatiently or with hostility toward colleagues or clients."*

**3. Reduced Personal Accomplishment (PA)**

This reversed subscale evaluates diminished self-efficacy and satisfaction related to decision

outcomes. Items may include:

- *"I feel increasingly less fulfilled by my work achievements."*

Interpretation:

EE high (≥12): Indicates severe emotional exhaustion and potential burnout.

DP high (≥6): Suggests detachment or impersonal attitudes toward others.

PA low (≤3): Suggests reduced feelings of personal achievement.

**3. Minnesota Satisfaction Questionnaire (MSQ) – Short Form**

Participants rated their satisfaction on a 5-point Likert scale (1 = Very dissatisfied, 5 = Very satisfied) across 20 items:

Intrinsic Satisfaction

1. Keeping busy all the time.

2. Opportunity to work independently.

3. Doing different things from time to time.

4. Being an important member of a group.

5. Doing things that do not go against my conscience.

6. Job stability.

7. Opportunity to help others.

8. Opportunity to use my abilities.

9. Freedom to make decisions.

10. Freedom to decide how to do my work.

11. Sense of achievement from work.

Extrinsic Satisfaction

12. The way my boss treats subordinates.

13. My supervisor’s decision-making ability.

14. Telling others what to do.

15. Company policy implementation.

16. Pay relative to workload.

17. Opportunities for promotion.

18. Working conditions.

19. Relationships with colleagues.

20. Rewards for good performance.

Interpretation:

Intrinsic Satisfaction low (<28): Low satisfaction with intrinsic aspects of work.

Extrinsic Satisfaction low (<23): Low satisfaction with extrinsic aspects of work.

**4. Connor-Davidson Resilience Scale (CD-RISC)**

Participants rated their agreement with the following statements on a 5-point Likert scale (0 = Not true at all, 4 = True nearly all the time):

1. I am able to deal with stress and challenges in life.

2. I can stay calm when things get tough.

3. I believe I can control many things in my life.

4. I remain optimistic about the future.

5. I can bounce back after setbacks.

6. I remain steady under pressure.

7. I can find support during difficult times.

8. I can set and achieve goals.

9. I find meaning and purpose in life.

10. I can cope with changes in life.

Interpretation:

Low resilience (<20): May benefit from psychological support and interventions.

Moderate resilience (21–30): Shows some resilience but may need strengthening in certain areas.

High resilience (>30): Indicates strong capacity to handle stress and challenges effectively.
